# Rye B chromosomes differently influence the expression of A chromosome–encoded genes depending on the host species

**DOI:** 10.1007/s10577-022-09704-6

**Published:** 2022-07-04

**Authors:** Anastassia Boudichevskaia, Anne Fiebig, Katrin Kumke, Axel Himmelbach, Andreas Houben

**Affiliations:** 1 Leibniz Institute of Plant Genetics and Crop Plant Research (IPK) Gatersleben, 06466 Seeland, Germany; 2 KWS SAAT SE & Co. KGaA, 37574 Einbeck, Germany

**Keywords:** Selfish B chromosome, RNA-seq, Comparative transcriptome analysis, Chromosome drive, Plants

## Abstract

The B chromosome (B) is a dispensable component of the genome in many species. To evaluate the impact of Bs on the transcriptome of the standard A chromosomes (A), comparative RNA-seq analyses of rye and wheat anthers with and without additional rye Bs were conducted. In both species, 5–6% of the A-derived transcripts across the entire genomes were differentially expressed in the presence of  2Bs. The GO term enrichment analysis revealed that Bs influence A chromosome encoded processes like “gene silencing”; “DNA methylation or demethylation”; “chromatin silencing”; “negative regulation of gene expression, epigenetic”; “post-embryonic development”; and “chromosome organization.” 244 B chromosome responsive A-located genes in + 2B rye and + B wheat shared the same biological function. Positively correlated with the number of Bs, 939 and 1391 B-specific transcripts were identified in + 2B and + 4B wheat samples, respectively. 85% of B-transcripts in + 2B were also found in + 4B transcriptomes. 297 B-specific transcripts were identified in + 2B rye, and 27% were common to the B-derived transcripts identified in + B wheat. Bs encode mobile elements and housekeeping genes, but most B-transcripts were without detectable similarity to known genes. Some of these genes are involved in cell division-related functions like Nuf2 and might indicate their importance in maintaining Bs. The transcriptome analysis provides new insights into the complex interrelationship between standard A chromosomes and supernumerary B chromosomes.

**Supplementary Information:**

The online version contains supplementary material available at 10.1007/s10577-022-09704-6.

## Introduction

The B chromosome (B) is one of the most interesting nonessential components of the genome in a wide range of taxa, from fungi to plants and animals. Although dependent on the species, Bs may vary in copy number, size, and structure; they share features that make them distinguishable from other types of chromosome polymorphisms like, for example, aneuploidy (reviewed in, e.g. Camacho et al. 2000; Douglas and Birchler, 2017; Houben et al. 2013; Jones 1995). Because most Bs do not confer any advantages on the host organisms that harbour them, they are considered as parasitic, selfish elements that persist in populations by making use of the cellular machinery required for the inheritance and function of standard chromosomes, which are also called A chromosomes (As). The presence of a low number of Bs is associated with mild or no apparent phenotypes. However, an increased number of Bs causes phenotypic differences and reduced fertility (reviewed in (Bougourd and Jones 1997).

Cultivated rye (*Secale cereale* subsp. *cereale*) tolerates up to eight Bs (Jones and Rees 1982). The number of Bs is stable in all somatic plant tissues, and each B of rye adds ~ 580 Mbp DNA to the standard complement containing seven pairs of A chromosomes (1C ~ 7917 Mbp). The rye B originated approximately 1.1–1.3 million years ago, 0.4–0.6 million years after the formation of the *Secale* genus (Martis et al. 2012). This supernumerary chromosome is likely a by-product of host genome evolution because the B shows extended sequence similarity with thousands of genic sequences derived from all A chromosomes as well as mitochondria and chloroplast genomes of rye. It seems that the B acts like a “sponge” that collects A chromosome-, chloroplast-, and mitochondrion-derived sequences. Almost proportional to the number of additional Bs, rye plants were reduced in weight, height, seed weight, and tiller numbers (Moss 1966; Müntzing 1963). Thus, under normal growth conditions, the existence of rye Bs is not beneficial for the host. However, rye plants with Bs showed heat-induced positive effects indicating that Bs have implications on heat tolerance during the early stages of male sporogenesis (Pereira et al. 2017).

To determine the transcriptional activity of the rye B, first, a cDNA AFLP analysis (Carchilan et al. 2009) and a comparative RNA-seq analysis were performed with mRNA samples of rye with and without Bs (Ma et al. 2017). In total, 1954 and 1218 B-located and expressed genic sequences with an open reading frame of ≥ 180 bp in generative and vegetative tissues were considered as B-located genic sequences (Ma et al. 2017). A detailed analysis of selected B-encoded genic fragments revealed alternative splicing expression patterns and the ability to modulate the expression of their counterparts on A chromosomes in a tissue‐ and genotype‐dependent manner, suggesting that at least for some B-located genic sequences, the regulatory elements remained functional (Banaei-Moghaddam et al. 2013). A similar transcription and regulatory behaviour have been demonstrated for the B chromosome of maize (Huang et al. 2016; Shi et al. 2022). However, the influence of the rye B on the rye host transcriptome and its impact on an alien host genome like wheat at the genome level has not been analysed yet.

One of the rye B-specific transcripts showed homology to Argonaute 4 of *Arabidopsis thaliana* (ScAGO4B). Sequence alignment of ScAGO4B showed that compared to the A-originated transcript, the B-transcript had minor changes, and none of the point mutations caused a non-sense mutation. In vitro analysis of the A and B chromosome encoded ScAGO4 variants demonstrated that both proteins retain RNA slicer activity (Ma et al. 2017). To test whether the B chromosome affects the proteome of the host, comparative mass spectrometry was performed using the protein samples isolated from shoots of rye plants with and without Bs. 319 out of 16,776 quantified peptides were found in at least three out of five + B plants but not in 0B plants. 31 out of 319 peptides were identified as B-associated peptide features. Thus, the existence of Bs alters the composition of the proteome (Ma et al. 2021).

Here, to evaluate the impact of Bs on the transcriptome of the standard A chromosomes, we conducted a comparative analysis of rye with and without Bs. In addition, a wheat-rye B chromosome addition line (Endo et al. 2008) was used to evaluate the effect of a B chromosome which was added to a foreign host species. To ensure the comparability of the transcriptomes, we selected in both species anthers undergoing the first pollen mitosis for comparative RNA-seq. However, although the rye B is of monophyletic origin and a comparative analysis of Bs of different rye genotypes showed at the cytological level high similarity (Marques et al. 2013), at the sequence level, differences likely exist between the rye Bs in the background of wheat and rye. However, as both genotypes were individually propagated over many generations, the Bs within the analysed wheat and rye genotypes are probably identical.

Our comparative RNA-seq analysis revealed that the presence of rye B chromosomes modulates the gene activity of the A chromosome complement of the natural host species rye and the alien host wheat. Thus, B encoded genes may provide an additional level of gene control and complexity in combination with coregulated A-located genes.

## Material and methods

### Plant cultivation and genotyping

Common wheat (*Triticum aestivum* L., cv “Chinese Spring”) with and without additional rye B chromosomes (Endo et al. 2008) were germinated under greenhouse conditions with a 16-h photoperiod (22 °C day/20 °C night, 50% relative humidity, 100 to 120 μmol m^−2^ s^−1^, light intensity) at the IPK, Gatersleben (Germany). The B chromosome of the wheat-rye B addition line was initially introduced into a Nepalese strain of wheat from a spring rye variety from Transbaikal, Siberia (Lindström 1965) and then transferred into the wheat genotype’Chinese Spring’ by Endo et al. (2008).

Rye (*Secale cereale* L.) plants of the Japanese JNK strain (Ribeiro et al. 2004) without and with additional rye Bs were initially grown under long-day conditions of 16 h light at 20 °C and 8 h dark at 18 °C, 100 to 120 μmol m^−2^ s^−1^ light intensity. At the fifth leaf stage, they were cultivated at 4 °C for additional two months. The number of Bs in root meristems was determined by fluorescence in situ hybridization (FISH), according to Klemme et al. (2013). For the identification of B chromosomes, the rye B-specific repeat D1100 (Sandery et al. 1990) was used as FISH probe. The probe was labelled by nick translation with ChromaTide Texas Red-12-dUTP (Molecular Probes; http://www.invitrogen.com).

### RNA isolation of staged anthers and Illumina sequencing

Anthers from five rye plants with 0B or + 2B were pooled to produce a respective biological replicate. For wheat, additionally, anthers were collected from five + 4Bs plants per replicate. For the RNA-seq analysis, four biological replicates per sample type were tested in wheat, and three biological replicates per condition were used in rye. To ensure the same developmental stage, only anthers at the first pollen grain mitosis were harvested, immediately frozen in liquid nitrogen and stored at − 80 °C until RNA extraction.

Total RNA was isolated from 50 mg anthers per sample using the plant/fungi total RNA purification kit (Norgen Biotek Corp, Thorold, ON, Canada) according to the manufacturer’s manual. RNA samples were treated with DNA-free DNase before cDNA synthesis following the on-column DNA removal protocol (RNase-Free DNase I Kit, Norgen Biotek Corp). The RNA concentration was determined using a Qubit device (RNA HS assay kit, Thermo Fisher Scientific Inc, Waltham, MA, USA). RNA Integrity Number (RIN) was verified using a 2100 Bioanalyser (Agilent, Santa Clara, CA, USA). Library preparation (Illumina TruSeq RNA Sample Preparation Kit) and sequencing-by-synthesis using the Illumina HiSeq 2500 device (DNA Sequencing Service of the IPK Gatersleben, Germany) involved standard protocols from the manufacturer (Illumina, Inc., San Diego, CA, USA). The libraries were quantified by qPCR (Mascher et al. 2013) and sequenced using the rapid run mode (paired-end, 2 × 101 cycles).

### Data preprocessing

All reads were preprocessed for quality control with FastQC (Galaxy v0.72, (Andrews, 2010). After read quality inspection, the Trimmomatic program (Galaxy v0.36.6 (Bolger et al. 2014)) was applied to filter out adaptors and low-quality sequences. Trimmed reads of replicates were combined into 0B and + 2B rye datasets for comparison of genomic backgrounds (Supplemental Dataset 1). In wheat, the same procedure led to 0B, + 2B, and + 4B wheat datasets (Supplemental Table 1).

### RNA-seq data processing to identify wheat host transcripts formed in the response of additional rye Bs

The trimmed reads of all samples (wheat 0B, + 2B, + 4B) were aligned against *Triticum aestivum* (IWGSC_Refseq v1.1) genome using the HISAT2 Galaxy tool v2.0.3 with default settings (Kim et al. 2015). HISAT2 generated two output files: aligned and unaligned mapping reads (Supplemental Table 1). The aligned output files from conditions wheat 0B and + 2B were used to identify transcripts derived from the standard A chromosomes of wheat. Read counts for each representative transcript were quantified based on the BAM files produced with the HISAT2 by using the tool feature Counts, Galaxy v1.4.6.p5 (Liao et al. 2014)*.* Differentially expressed features were determined based on the feature Counts tables by applying the tool DESeq2, Galaxy v2.11.38 with the setting parameter fit type = “parametric” (Love et al. 2014). DESeq2 tested for differential expression is based on a model using the negative binomial distribution (Love et al. 2014). Differentially expressed transcripts were identified by comparison of wheat 0B vs wheat + 2B. The results of the statistical test were adjusted for the multiple testing FDR with the Benjamini and Hochberg procedure (Benjamini and Hochberg 1995). A cutoff value of adjusted *P* values equal to 0.05 was chosen as the threshold to identify significant differentially expressed transcripts.

### RNA-seq data processing to identify rye host transcripts formed in the response of additional rye Bs

The trimmed reads of all rye 0B and + 2B samples were aligned against the *S. cereale* genome (*Secale_cereale*_Lo7_2018v1p1p1_pseudomolecules, February 2019, GCA_902687465.1) using HISAT2 v2.1.0 with default settings. The generated by HISAT2 aligned mapping reads were proceeded further with the pseudo-alignment Kallisto (v0.44.0)(Bray et al. 2016) to count reads (est counts) at the transcript level. To identify differentially expressed transcripts (DETs), the DESeq2 analysis was carried out using the Trinity pipeline (v2.6.5) with the threshold FDR (False Discovery Rate) of 0.05. The data quality based on Pearson’s correlation and principal component analysis (PCA) were estimated with “PtR” of the Trinity pipeline (Supplemental Dataset 2).

### RNA-seq data processing to identify transcripts derived from the rye Bs in the background of wheat

Reads (wheat 0B, + 2B, + 4B) which did not align against the *T. aestivum* (IWGSC_Refseq v1.1) genome using the HISAT2 Galaxy tool v2.0.3 (Kim et al. 2015), with default settings were used as input for the de novo transcriptome assembly based on Trinity v2.6.5 (Grabherr et al. 2011). 0B, 2B, and 4B datasets were separately assembled. Trinity results provided a set of sequences (called “isoforms”) grouped into clusters. The quality and completeness of assemblies were determined using TransRate v1.0.3 (Smith-Unna et al. 2016), which resulted in optimized assemblies of 0B, 2B, and 4B containing 13,940, 16,414, and 13,068 contigs, respectively. To track the origin of contigs, the sequences were renamed with 0B, 2B, and 4B prefixes for each contig. In addition, three files plus cDNA from *T. aestivum* (IWGSC, EnsemblePlants release 42) were catenated into one dataset and further processed by CD-HIT (CD-HIT-EST) v4.6.8 (sequence identity threshold 0.90) (Fu et al. 2012; Li and Godzik 2006) to reduce redundancy and cluster highly homologous sequences. The final file was used for Kallisto indexing and read counting (v0.44.0) (Bray et al. 2016). log_2_-transformed counts-per-million (CPM) values were generated and “PtR” of the Trinity pipeline was used to examine the data quality based on Pearson’s correlation. The final FASTA file with contigs starting with 2B and 4B prefixes was used as a reference file in mapping of rye samples and identification of B-specific reads (see below). B-specific transcripts were filtered based on a minimal expression of 0.1 TPM in all replicates representing 2B and 4B conditions, respectively, and a maximal expression of 0.09 TPM in all replicates representing 0B condition. Additionally, only transcripts with prefixes 2B and 4B were considered. The principal component analysis was done in DESeq2 (Supplemental Dataset 3).

### RNA-seq data processing to identify transcripts encoded by the rye B in the background of rye

The trimmed reads of rye 0B and + 2B samples were mapped against the rye B transcriptome identified in the previous step in wheat carrying Bs using HISAT2 v2.1.0 with default settings. The HISAT2-based aligned mapping reads were proceeded further to quantify the abundances of transcripts with Kallisto (v0.44.0) (Bray et al. 2016). log_2_-transformed counts-per-million (CPM) values were generated, and “PtR” of the Trinity pipeline was used to examine the data quality based on Pearson’s correlation (Supplemental Fig. 1). Based on the Kallisto procedure, B-specific transcripts were identified by applying a threshold for a minimal expression is 0.1 TPM in all replicates representing a 2B condition and a maximal expression is of 0.09 TPM in all replicates representing a 0B condition.

### Transcriptome annotation of B-specific transcripts

Transdecoder v5.3.0 (http://transdecoder.github.io) was used to predict open reading frames (ORFs) in the B-specific transcriptome, which were annotated by Interproscan Galaxy v5.0.2 mkh. Additionally, sequences were BLASTed against the wheat genome IWGSC Ref.Seq.1.1 using *E*-values < 1.0E − 3 in at least 100 amino acids.

### BLASTP of differentially expressed transcripts of rye

For further classification of differentially expressed transcripts identified as a rye host response in the presence of additional rye Bs, DETs were BLASTed (BLASTP) against the wheat genome IWGSC Ref.Seq.1.1 using a bit score at least 50 in at least 100 amino acids using the first best hit criterion. The annotated DETs were used in downstream GO term enrichment analysis.

### GO Term enrichment analysis

To determine classes of genes that are over-represented in a large set of genes and may have an association with the presence of B chromosomes in wheat and rye, the Gene Set Enrichment Analysis tool (GSEA, AgriGO v2. analytical toolkit (Tian et al. 2017) was used. The enrichment analysis is a systematic approach for interpretation of the candidate genes extracted from the RNA-seq or other high-throughput biotechnologies. In this study, only significantly differentially transcribed genes with a cut-off of |log_2_(fold change) |≥ 0.5 and adjusted *P* < 0.05 were selected. Gene-specific *P* values were obtained from the hypergeometric test and were further corrected via Hochberg FDR option. A FDR < 0.05 cut-off has been set for detecting significantly enriched groups. For the GO term enrichment analysis, the separated analyses of upregulated and downregulated transcripts were performed. It was demonstrated that the analysis of all DE genes together could greatly reduce the power to identify significant pathways due to the imbalance between the upregulated and downregulated genes. In wheat and rye, biological processes (BP), molecular functions (MF), and cellular components (CC) were assessed using *T. aestivum* (“Chinese Spring”) as a background reference since the wheat is better annotated and described than rye. GO term enrichment analysis results were visualized with REVIGO (Supek et al. 2011). Similar GO terms are grouped based on semantic similarity. Statistical enrichment analysis for orthologue differentially expressed genes (DEGs) was performed with the g:profiler webserver (Raudvere et al. 2019) using wheat as a reference. A FDR < 0.05 cut-off has been set for detecting significantly enriched groups.

### Plotting gene density and orthologous genes

Gene density for DEGs of rye and wheat + 2B conditions and the overall protein-coding gene density were calculated using a sliding window of 50 Mbp. Two BED files were used containing the chromosomal coordinates of each reference genome (*T. aestivum* Refseq_1.0 and *S. cereale* Lo7 2018v1p1p1) as well as the coordinates of (i) upregulated and downregulated DETs and (ii) all protein-coding features. Overlaps were identified and counted using the bedops suite (v2.4.19) (Neph et al. 2012).The distribution of gene counts along the wheat and rye chromosomes is plotted with karyoplotteR (v1.20.2) (Gel and Serra 2017).To infer ontology between DEGs of host genomes under + B conditions in (i) wheat and rye and (ii) among wheat subgenomes (A, B, and D), reciprocal best hits (RBH) were used to identify pairs of highly similar genes. Reciprocal BLASTP searches were computed for all sets with the following option: -e-value = 1e − 50. Hits were prefiltered for a minimum of 50% identity. Final RBHs were selected using a custom bash script. Pairwise hits were ranked based on E-value and percental identity to find pairs among the highest scoring genes. In case of multiple identical hits (among wheat subgenomes), all pairs were kept for further analysis. The R package VennDiagram (v1.7.3) (Chen and Boutros 2011) was used to depict the results of both comparisons.

### Data availability

Raw sequence reads can be obtained from the European Nucleotide Archive (ENA) under the study accession number ID PRJEB46034, available at https://www.ebi.ac.uk/ena/browser/view/PRJEB46034.

## Results

### The transcriptomes of rye and wheat standard A chromosomes are differentially affected by the presence of rye B chromosomes

**Fig. 1:**
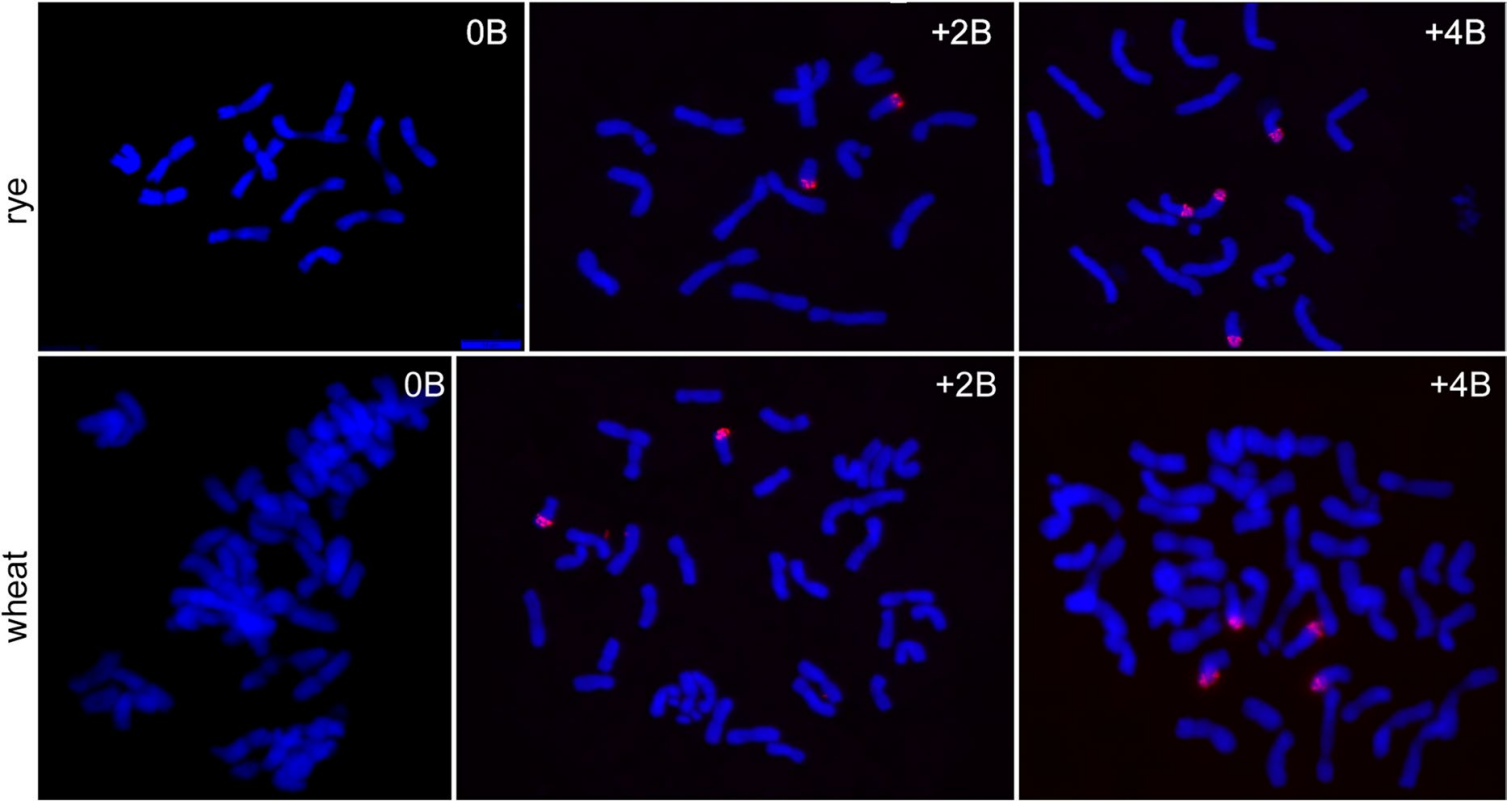
Mitotic metaphase cells of rye and wheat possessing additional rye B chromosomes (0B, + 2B, + 4B). Bs were identified by FISH using the B-specific repeat D1100 (in red). Chromosomes were counterstained with DAPI

To study whether rye B chromosomes influence the transcriptome of rye and wheat standard A chromosomes, first, we identified 0B and + B containing plants by FISH using the repeat D1100 (Sandery et al. 1990) as a B-specific probe (Fig. 1). After, the total RNA was extracted from anthers undergoing the first pollen mitosis of both species with and without Bs. Anthers from rye with 0B or + 2B were pooled to produce a respective biological replicate. For wheat, anthers of plants with 0B, + 2B, or + 4B were collected. For RNA-seq analysis, four and three biological replicates per sample type were used in wheat and rye, respectively.

**Fig. 2:**
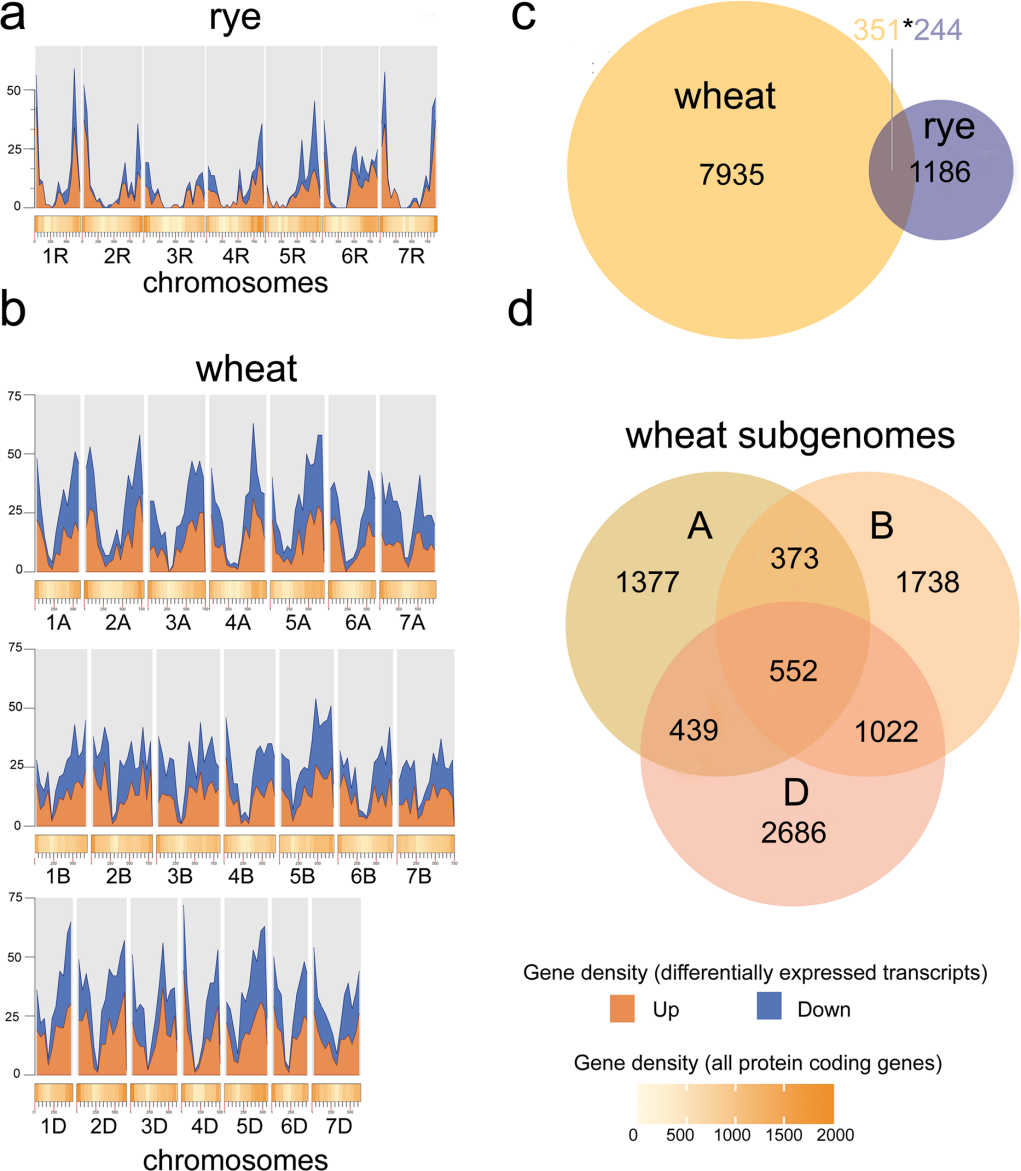
Density and chromosomal distribution of all host genome located protein-coding genes and DEGs **a** in rye with + 2B and **b** in wheat with + 2B. Upregulated and downregulated DEGs are shown in blue and orange, respectively. **c** Comparison of orthologous DEGs identified in the host genomes based on identical reciprocal best hits (RBHs) of wheat + 2B (total number 8286) and rye + 2B (total number 1430). 351 in wheat-derived DEGs are shared with in rye identified DEGs. 244 in rye-derived DEGS were shared with in wheat identified DEGs. **d** Comparison of orthologous DEGs identified in the wheat + 2B subgenomes A, B, and D (total number 8187). Pairs of genes sharing the same functionality are inferred from reciprocal best hits BLAST

High-quality RNA-seq reads of 0B and + 2B rye anthers were mapped onto the reference rye genome for the comparative transcriptome analysis, and the overall alignment rate for all samples was 75% (Supplemental Dataset 1). 1430 (5.5%) of in total 25,973 A chromosome-derived transcripts were significantly differentially expressed after comparison of 0B and + 2B conditions using a DESeq2 and the false discovery rate < 0.05. The majority (1286) of differentially expressed transcripts (DETs) was highly differentially expressed (log_2_ Fold Change (FC) ≥ 1). Among highly DETs, 864 were activated in the presence of + 2B and 424 strongly decreased their expression (Supplemental Fig. 2a, Supplemental Table 2). 19.5% of DETs (279) showed a very high expression level (FC > 32, Supplemental Tables 2–3). Corresponding upregulated and downregulated rye genes are distributed on all standard A chromosomes (Fig. 2a). Hence, the presence of Bs influences the transcriptome of the entire standard rye A chromosome complement.

To study whether the rye B chromosome also impacts the transcriptome of the standard A chromosomes if introgressed into another species, we compared the transcriptomes of wheat anthers with two and without rye Bs undergoing the same developmental stage, the first pollen mitosis. The overall alignment rate of mapped high-quality RNA-seq reads was over 80% (Supplemental Table 1). The presence of + 2B in wheat led to the transcriptome reprogramming of 8286 standard A chromosome-derived transcripts, which accounted for 6.2% of all analysed transcripts (133,744) (Supplemental Tables 4–5). 26.8% of DETs (2220) were highly differentially expressed (log_2_ FC ≥ 1), including 1366 upregulated and 854 downregulated transcripts (Supplemental Fig. 2b). In contrast to rye, the highest proportion of DETs included those with log_2_ FC < 1 (Supplemental Table 4). All three subgenomes of wheat (A, B, and D genome; 2741, 2760, and 2686 transcripts, respectively) and chromosomes contributed to differentially expressed genes. Thus, irrespectively of the host genome species, 5–6% of the standard A chromosome-derived transcripts of rye and wheat are affected by additional two rye B chromosomes. Among actively transcribed genes, there were those involved in chromosome, microtubule, or cell cycle-related processes (Table 1). However, no obvious phenotype effect of two Bs was found on the rye and wheat plants grown under standard conditions.

**Table 1:** A list of differentially expressed genes involved in chromosomes, microtubules, or cell cycle-related processes and detected in rye and wheat in the presence of B chromosomes. 0B condition was compared against 0B + 2B

Species	Gene Id	Protein	log_2_FC	FDR corrected *P*-value
Rye	SECCE1Rv1G0044750.1	Structural maintenance of chromosomes protein	− 1.91	0.02548
	SECCE2Rv1G0090620.1	TPX2 (targeting protein for Xklp2) protein family	− 5.54	3.6E − 115
	SECCE3Rv1G0201660.1	Mitotic spindle assembly checkpoint protein MAD1	1.06	0.005558
	SECCE4Rv1G0234990.1	Sister-chromatid cohesion protein 3	− 2.53	1.19E − 14
	SECCE4Rv1G0273150.1	Cell cycle regulated microtubule associated protein	− 4.38	1.78E − 20
	SECCE4Rv1G0286900.1	Structural maintenance of chromosomes family protein	− 2.99	0.008777
	SECCE5Rv1G0321180.1	Structural maintenance of chromosomes family protein	− 3.27	2.59E − 19
	SECCE5Rv1G0361110.1	Cell division cycle 5-related protein	1.12	0.008589
	SECCE6Rv1G0379220.1	Kinetochore protein Nuf2, putative	− 3.84	0.038123
	SECCE6Rv1G0381900.1	Kinetochore protein Nuf2	− 3.73	5.83E − 06
	SECCE6Rv1G0404290.1	Microtubule associated family protein	− 3.42	0.001597
	SECCE6Rv1G0404750.1	Cell division topological specificity factor	− 2.72	7.92E − 08
	SECCE6Rv1G0404790.1	Kinetochore protein	1.36	0.012086
	SECCE7Rv1G0469610.1	Cell division cycle protein 48-like protein	− 0.59	0.042261
	SECCE7Rv1G0514140.1	AUGMIN subunit 2	− 3.63	3.15E − 06
Wheat	TraesCS1A02G366800.1	Regulator of chromosome condensation (RCC1) family with FYVE zinc finger domain-containing protein	− 0.86	0.012119
	TraesCS1B02G235300.1	Cell division cycle 48-like protein	− 1.46	0.009394
	TraesCS1B02G384600.1	Regulator of chromosome condensation (RCC1) family protein	− 1.04	0.042168
	TraesCS1D02G372300.1	Regulator of chromosome condensation (RCC1) family protein	− 1.08	0.005614
	TraesCS2A02G246100.1	Ankyrin repeat family protein/regulator of chromosome condensation (RCC1) family protein	− 0.69	0.002897
	TraesCS2A02G417700.1	Histone H4	− 1.06	0.009229
	TraesCS2B02G270400.1	Ankyrin repeat family protein/regulator of chromosome condensation (RCC1) family protein	− 0.63	0.017481
	TraesCS2B02G478900.1	Regulator of chromosome condensation (RCC1) family with FYVE zinc finger domain-containing protein	0.71	0.030691
	TraesCS2D02G457000.1	Regulator of chromosome condensation (RCC1) family with FYVE zinc finger domain-containing protein	0.84	0.005557
	TraesCS3D02G154000.1	Cell division cycle 48-like protein	− 1.03	0.048785
	TraesCS4D02G302800.1	Ankyrin repeat family protein/regulator of chromosome condensation (RCC1) family protein	− 1.17	0.044619
	TraesCS4D02G303000.1	Ankyrin repeat family protein/regulator of chromosome condensation (RCC1) family protein	− 1.15	0.045249
	TraesCS5B02G301800.1	Histone H4	− 0.87	0.010615
	TraesCS5D02G193100.1	WD-repeat cell cycle regulatory protein	− 1.08	0.017086
	TraesCS5D02G354700.1	Histone H4	− 0.41	0.035056
	TraesCS6A02G159700.1	Regulator of chromosome condensation (RCC1) family protein	− 0.79	0.00232
	TraesCS6A02G231400.2	Sister chromatid cohesion protein PDS5 B-B	1.82	2.47E − 06
	TraesCS6B02G059400.1	Sister chromatid cohesion protein PDS5 like B-B	0.54	0.036613
	TraesCS6D02G142500.1	Regulator of chromosome condensation (RCC1) family protein	0.85	0.015492
	TraesCS6D02G241800.1	Regulator of chromosome condensation (RCC1) family with FYVE zinc finger domain-containing protein	1.05	0.013112

To identify genes expressed under + 2B conditions in the rye and wheat host genomes sharing the same biological function, pairs of genes based on reciprocal best hits (RBH) were computed. A total of 244 in rye identified DEGs are shared with the DEGs of the wheat host genome (Fig. 2c; Supplemental Table 6). 351 in wheat derived DEGs are shared with in rye identified DEGs. Furthermore, the grade of homology within the 8187 differentially expressed genes among the wheat subgenomes under der influence of rye B chromosomes was determined (Fig. 2d). A fraction of 552 (6.7%) genes are found in all subgenomes. 22% of all DEGs have homologue genes in two subgenomes (AB, AD, BC) with an overrepresentation in the BD group. 71% of DEGs in the wheat host genome do not share any homologies with other subgenomes.

### GO enrichment analyses reflect the impact of B chromosomes on the host genome

To explore the regulatory pathways of DETs identified as a host response, a GO enrichment analysis was performed with rye and wheat datasets with wheat as a background reference. Separate studies of upregulated and downregulated transcripts were carried out, and different enriched GO terms were found. The dataset of upregulated transcripts in + 2B rye was represented with 578 annotated genes. Biological processes (BP) were described with 110 GO terms. Among them, “negative regulation of gene expression, epigenetic”; “chromatin silencing”; and “chromosome organization” were the most statistically significant. Other overrepresented GO terms included “methylation,” “cell cycle process,” “chromatin organization,” and “mitotic cytokinesis.” In the molecular function (MF) category, there were 18 overrepresented terms with the most statistically significant term “choline dehydrogenase activity.” The cellular component (CC) category was represented with 50 terms. Among them, there were “chromosome,” “chromatin,” “microtubule,” and “chromosome, centromeric region.”

The dataset of downregulated rye A chromosome transcripts in the presence of 2B was represented with 363 annotated genes. The BP category was characterized with 89 terms; MF and CC categories included eight and 38 terms, respectively. The enriched GO terms of the BP category were related to metabolism, photosynthesis, and several kinds of responses. The most statistically significant terms of the MF were “catalytic activity” and “hydrolase activity.” The CC category included terms related to chloroplast and plastids (Supplemental Dataset 4). In the presence of  2B in wheat, activated transcripts accounted for 428 enriched BP terms, 159 CC terms, and 171 MF terms (Supplemental Dataset 5, sheet 1). GO terms as “translation,” “RNA methylation,” and “methylation” were among the most statistically significant biological processes. Other enriched GO terms referred to the biological processes such as “mitotic G2 phase,” “mitotic cell cycle phase,” “chromatin organization,” and “regulation of chromosome organization.”

The dataset of downregulated wheat transcripts in the presence of 2B was represented with 793 BP terms, 134 CC terms, and 204 MF terms (Supplemental Dataset 5, sheet 2). The most prominent GO term related to BP was “photosynthesis.” Other highly overrepresented GO terms described “metabolic processes,” “cellular processes,” “response to stress,” and “transcription.” According to enrichment analysis, the processes of seed germination, plant growth and development, leaf development, root morphogenesis, and reproduction could be affected in wheat because of the presence of 2B (Table 2). Gene set enrichment analysis in 0B/ + 2B rye and 0B/ + 2B wheat revealed common GO terms in both host species (Supplemental Dataset 6).

**Table 2:** Assignment of differentially expressed downregulated transcripts in wheat in the presence of 2B chromosomes to different functional categories related to plant growth and development. Gene Ontologies were analysed for term enrichment using the GSEA, AgriGO v2. analytical toolkit (cutoff of 5% false discovery rate (FDR))

GO Id	Biological process	Number in input list	Number in Ref	*P*-value	FDR rate
GO:0032502	Developmental process	973	23,141	9.2e − 09	8.6e − 08
GO:0030154	Cell differentiation	354	7284	1.1e − 08	9.7e − 08
GO:0048507	Meristem development	195	3564	1.7e − 08	1.5e − 07
GO:0040007	Growth	356	7381	2E − 08	1.8e − 07
GO:0035266	Meristem growth	123	2014	4.2e − 08	3.5e − 07
GO:0009965	Leaf morphogenesis	127	2118	6.5e − 08	5.2e − 07
GO:0048589	Developmental growth	295	6124	4.2e − 07	3.1e − 06
GO:0009845	Seed germination	113	1986	3.4e − 06	2.2e − 05
GO:0040008	Regulation of growth	184	3670	8.2e − 06	4.9e − 05
GO:0009932	Cell tip growth	117	2124	9.3e − 06	5.5e − 05
GO:0050793	Regulation of developmental process	299	6509	1.3e − 05	7.7e − 05
GO:0048518	Positive regulation of biological process	349	7797	1.9e − 05	0.00011
GO:0080147	Root hair cell development	68	1141	8.1e − 05	0.0004
GO:0048366	Leaf development	206	4388	0.0001	0.00047
GO:0048608	Reproductive structure development	472	11,435	0.00079	0.0031
GO:0010015	Root morphogenesis	168	3786	0.0039	0.012
GO:0048868	Pollen tube development	103	2182	0.0043	0.014
GO:0022414	Reproductive process	604	15,247	0.0044	0.014
GO:0048316	Seed development	277	6660	0.007	0.021
GO:0099402	Plant organ development	426	10,673	0.011	0.032
GO:1900140	Regulation of seedling development	55	1120	0.016	0.042

Total query of input is 3073 genes, and the total background number is 85,761 genes (reference wheat, “Chinese Spring”)

In the case of upregulated standard A chromosome transcripts, the BP was represented with 53 common terms, including “chromosome organization,” “gene silencing,” “regulation of chromatin organization,” “mitotic cytokinetic process,” and “methylation” (Supplemental Fig. 3a). The MF was included 11 common terms. CC category included 30 common terms, including “chromatin.” In the case of downregulated transcripts, the BP consisted of 74 common enriched terms, including “metabolic process,” “transport,” different kinds of responses, and “photosynthesis” (Supplemental Fig. 3b). Interestingly, the CC category (33 common terms), including chloroplast and plastid-related terms, suggests repression of photosynthesis-related processes. Since the enrichment analysis in rye was based on the wheat background reference, the number of common overrepresented terms can be higher. Hence, the rye B chromosome impacts the A-located genes of rye and wheat in diverse biological processes.

### Identification of rye B chromosome-specific transcripts in the background of wheat

After the identification of A chromosome-responsive genes, we aimed for the identification of rye B-specific transcripts. The high sequence similarity of A and B chromosomes of the same species is challenging the transcription analysis of Bs. To overcome this problem, we characterized rye B-specific transcripts in the background of wheat first. The starting material for the de novo assembly was those reads that were not mapped against the *T. aestivum* (IWGSC_Refseq v1.1) genome. To identify rye B-specific transcripts, RNA-seq data from wheat with and without Bs were compared. The de novo transcriptome assembly followed by filtering and clustering led to the final FASTA file with 8611 transcript sequences specific to + 2B and/or + 4B transcriptomes (Supplemental Fig. 4).

Both B chromosome transcriptome profiles were evaluated at the transcript isoform expression level. A correlation matrix and principal component analysis (PCA) confirmed biological replications (Supplemental Dataset 3). As a result, 939 transcripts (10.9%) were identified in the + 2B sample, 1391 (16.2%) transcripts in the + 4B condition, and 85% of B-transcripts in + 2B were also found in the + 4B transcriptome.

For the functional annotation, only assembled transcripts with an open reading frame of ≥ 100 AA were considered. Like reported before (Ma et al. 2017), the largest part of the B-specific transcripts is without detectable similarity to functionally annotated proteins. In the + 2B transcriptome, only 23.6% of all B-specific transcripts were annotated, and in + 4B transcriptome, there were 13.4% annotated transcripts. In the list of + 2B and + 4B transcripts, 187 transcripts were annotated (23.6%, Supplemental Dataset 7).

Among annotated transcripts common to + 2B and + 4B samples, in line with other studies showing that transposable elements are a major component of the B chromosome of rye and other species (Blavet et al. 2021; Carchilan et al. 2009; Coan and Martins 2018; Klemme et al. 2013; Shi et al. 2022), we found transcripts encoding transposable elements (MuDR transposase, MULE transposase, retrotransposon Gag protein). In addition, cell cycle-related B-specific transcripts were identified like encoding the kinetochore protein Nuf2, kinesins (KIN4C, KIN6, KIN 10B), a DEAD/DEAH box helicase, and the microtubule-associated protein (TPX2).

### Identification of rye B-chromosome specific transcripts in the background of rye

The final FASTA file with + 2B and + 4B specific transcripts (8611) in the background of wheat was used as a reference to map B chromosome-specific reads in rye (Supplemental Fig. 4). As a result, 297 B-specific transcripts were confirmed as B-specific in rye + 2B. The number of B-specific transcripts is likely underestimated due to the high similarity of B transcripts to the rye host sequences. 27% were common to the + 2B and + 4B transcripts identified in wheat. Of 297 B-specific transcripts identified in the rye, 36 of them were annotated (Supplemental Dataset 7). Like in the case of + 2B and + 4B specific transcripts identified in wheat, there were also transcripts encoding transposable elements and reverse transcriptases. Among the common annotated transcripts, there were transcripts encoding the cell cycle-regulated microtubule-associated protein (TPX2), the SWIM zinc finger domain, a XPC-binding domain, and an IQ calmodulin-binding motif.

## Discussion

**Fig. 3:**
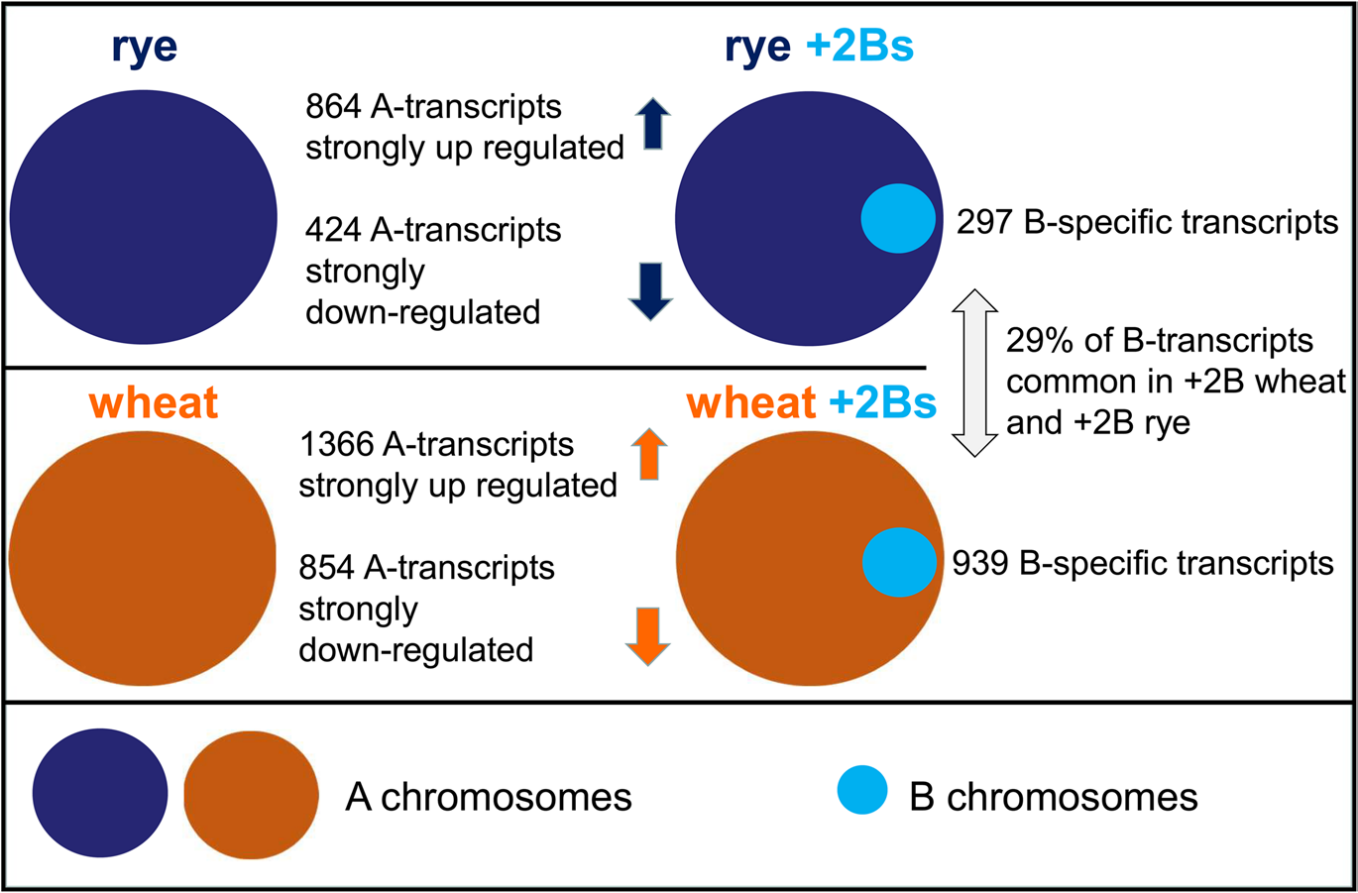
The transcriptional effect of rye B chromosomes in the background of rye and wheat. The occurrence of differentially expressed transcripts identified as a rye and wheat host response in the presence of additional rye B chromosomes. In addition, the number of rye B-specific transcripts determined in both species is shown

As summarized in Figure 3, we studied the transcriptomes of wheat and rye anthers with and without rye B chromosomes to determine the impact of the B on the gene transcription of standard A chromosomes in rye and wheat. In addition, B chromosome-specific transcripts in the background of both species were analysed. Our experimental set-up allowed a comparative transcriptome analysis of 0B/ + 2B rye and 0B/ + 2B wheat anthers and the identification of comprehensive changes in the expression of standard A chromosome-located genes due to the presence of rye Bs. We applied a set of strict criteria to distinguish A and B derived transcripts in both host species. In order to ensure the correct selection of B-derived transcripts, we employed the comparative 0B/ + B wheat transcriptomes first, because of the higher sequence dissimilarity between wheat and rye. B-specific transcriptome identified in the background of wheat allowed the dissection of B-specific transcripts in the rye host.

In wheat and rye, 5–6% of the standard A chromosome-derived transcripts are affected by additional 2Bs of rye. Strikingly, depending on the host species, the presence of rye Bs affects the expression of A chromosome–encoded genes differently. In rye, most differentially expressed transcripts (DETs) were highly differentially expressed, while in wheat, the highest proportion of DETs included those with log_2_ FC < 1.

The reason behind the host species depending response of rye Bs is likely complex. The following factors could play a role; (I) the genome size and ploidy differ (diploid versus hexaploid) between both species. As suggested by Blavet et al. (2021), the effects of Bs on the host plant depend on the proportion of their DNA in the nucleus. According to earlier research, reductions in plant fitness are pronounced when the B chromosome mass exceeds 20% of DNA in the nucleus. Therefore, a higher number of A chromosome genes revealed mainly weak (log_2_ FC < 1) B-responsive transcription in hexaploid wheat. (II) The rye B in the background of wheat could be considered as an alien chromosome, while the B of rye is part of the rye pangenome. During species evolution, the interaction between A and B chromosomes of rye likely coadapted. It is likely that Bs may bring about various epigenetic effects, including the differential regulation of A-localized transposable elements through mechanisms such as homology-dependent RNA interference pathways. Since the rye B likely originated from the rye A chromosome complement, it seems reasonable to suggest that the transcription alterations of A-located sequences are caused by homology-dependent mechanisms, as has been proposed for the remodelling of gene activities after chromosome segment duplications (Shi et al. 2020). (III) Another hypothesis for explaining how the Bs exert control over the rest of the genome postulates their effects on the spatial organization of the genome itself (Margueron and Reinberg 2010). Although the general interphase structure does not differ between rye and wheat, the nuclear volume of both species differs. (IV) Although the rye B is of monophyletic origin (Niwa and Sakamoto 1995), the composition of rye Bs in the background of rye and wheat likely changed at the sequence level over generations and at least some differentially expressed genes could be explained. A comparative analysis of rye and wheat possessing the very same B would be ideal, but such material does not exist.

A comparable transcription effect of Bs was found in maize (Huang et al. 2016; Shi et al. 2022). Also, in *Lilium amabile* plants, recent RNA-seq analysis from leaf tissues of 0B and + 1B chromosomes revealed that 5.1% of the total transcripts were differentially expressed (threshold log_2_FC >|2|) (Park et al. 2019). Notably, an almost similar change of the total transcriptome was reported for plants carrying additional standard A chromosomes derived from a different species. For example, for the wheat — barley 7HL addition line 3% DEGs were found (Dong et al. 2020; Rey et al. 2018), and the analysis of wheat — *Aegilops longissima* 6Sl and 3Sl addition lines identified 4.3 to 5.4% DEGs (Dong et al. 2020; Rey et al. 2018).

Despite that a low number of Bs does not change notably the plant phenotype, based on our GO term enrichment analysis, we noted an impact of the Bs on the host transcriptome involved in processes like seed germination, plant growth and development, leaf development, root morphogenesis, and reproduction. Whether the decreased fitness of plants with a higher dosage of Bs can be explained by the B-trigged expression of such genes needs to be verified. In addition, GO analysis in wheat and rye revealed enrichment for genes in cellular processes. In the presence of rye B, such processes as “chromosome organization,” “cellular component assembly,” “regulation of chromatin organization,” and “mitotic cytokinetic process “ were significantly overrepresented in both host species indicating the strategy of B chromosomes to transmit during the cell division.

The lower number of B*-*specific transcripts in the background of wheat (939 transcripts in 2B) and rye (297 transcripts in + 2B) identified in our study compared to 1569 transcripts in Ma et al. (2017) can be explained by several reasons. (I) We used a different strategy for the identification of the unique B transcripts. (II) We had a higher number of biological replicates and anthers at a defined developmental stage. By setting a threshold, we considered all replicates. (III) For annotation with Interproscan, the threshold in our study was 100 AA minimum for the length of the sequence. In Ma et al. (2017), the length was 60 AA.

A B chromosome-specific response of cell division-related genes was found. These were transcripts encoding the kinetochore protein Nuf2 and kinesins. Alteration of Nuf2 in *A. thaliana* affects the microtubule organization and results in lagging chromatids (Li et al. 2021). Interestingly, B-specific Nuf2 transcripts are also associated with the process of programmed elimination of B chromosomes in *Aegilops speltoides* embryos (Boudichevskaia et al. 2020). *Ae. speltoides* is a related species of wheat and rye. It is tempting to speculate that the B-encoded Nuf2 variant is involved in the process of B chromosome nondisjunction. Nondisjunction of Bs is a key component of both processes, chromosome drive, and chromosome loss (Ruban et al. 2020). B chromosomes of other species also encode kinetochore genes. The Bs of the Cichlid fishes *Astatotilapia latifasciata* and *Metriaclima zebra* encode kinetochore-associated SKA (Valente et al. 2014) and KNTC1 (Clark et al. 2018) proteins, respectively. The B chromosomes of rye (our study) and *Astatotilapia latifasciata* (Valente et al. 2014, reviewed in Ahmad and Martins, 2019) expressed microtubule-associated TPX2-like transcripts. DEAD/DEAH box helicase-like transcripts were also identified for the Bs of rye (our study) and maize (Huang et al. 2016). Whether these genes are involved in the regulation of the B chromosome dive remains an intriguing question. The DEGs from the current analysis will provide a valuable resource for studying the impact of B chromosomes on the transcriptome of the A chromosomes complement and plant evolution. Considering the effect of Bs on the transcriptome of the host genome also suggests that care should be taken if B chromosome-based mini-chromosomes are used as a vehicle to transfer gene complexes in future.

## Supplementary Information

Below is the link to the electronic supplementary material.

Supplemental Dataset 1 RNA-seq quality: Rye samples. (XLSX 21 KB)

Supplemental Dataset 2 PCA plots based on the “PtR” of the Trinity pipeline. Pearson’s replicate correlation heatmap. (DOCX 88 KB)

Supplemental Dataset 3 Correlation of four biological replicates of rye B specific transcripts derived from wheat +2B plants. Correlation of four biological replicates of rye B specific transcripts derived from wheat +4B plants. PCA based on four biological replicates of rye B specific transcripts derived from wheat +2B and wheat +4B plants. (DOCX 73 KB)

Supplemental Dataset 4 GO term enrichment analysis of up-regulated and down-regulated DETs of rye in the presence of +2B chromosomes. T. aestivum is the background reference. (XLSX 29 KB)

Supplemental Dataset 5 GO term enrichment analysis of up-regulated and down-regulated DETs of wheat in the presence of +2B chromosomes. T. aestivum is the background reference. (XLSX 121 KB)

Supplemental Dataset 6 Common overrepresented GO terms identified in up-regulated and down-regulated DETs in rye and wheat in the presence of +2B chromosomes. T. aestivum is the background reference. (XLSX 30 KB)

Supplemental Dataset 7 Rye B specific transcripts identified in wheat and rye and annotated based on the Interproscan and BLASTP against T. aestivum. (XLSX 2556 KB)

Supplementary file8 (DOCX 36 KB)

Supplementary file9 (PNG 31 KB)

Supplementary file10 (PNG 38 KB)

Supplementary file11 (PPTX 620 KB)

Supplementary file12 (PPTX 71 KB)

Supplemental Table 1 RNA sequencing and read mapping onto the T. aestivum reference genome. (XLSX 11 KB)

Supplemental Table 2 Differentially expressed transcripts identified in rye in the presence of +2B chromosomes. (XLSX 9 KB)

Supplemental Table 3 Annotated differentially expressed transcripts in rye in the presence of +2B chromosomes. (XLSX 182 KB)

Supplemental Table 4 Differentially expressed transcripts identified in wheat in the presence of +2B chromosomes. (XLSX 10 KB)

Supplemental Table 5 Annotated differentially expressed genes identified in wheat in the presence of +2B chromosomes based on the DESeq2. (XLSX 1270 KB)

## Abbreviations

AA: Amino acid
AFLP: Amplified fragment-length polymorphism
As: A chromosomes
Bs: B chromosomes
BP: Biological processes
cDNA: Complementary DNA
CC: Cellular components
CPM: Counts-per-million
DETs: Differentially expressed transcripts
DEGs: Differentially expressed genes
FC: Fold change
FDR: False discovery rate
GO: Gene Ontology
GSEA: Gene set enrichment analysis
h: Hour
Mbp: Mega base pairs
MF: Molecular functions
mRNA: Messenger RNA
ORFs: Open reading frames
PCA: Principal component analysis
RBH: Reciprocal best hits
RNA-seq: RNA sequencing
TPM: Transcript per million
2Bs: 2B chromosomes
4Bs: 4B chromosomes
