# Supplementary material for: Rye B chromosomes differently influence the expression of A chromosome–encoded genes depending on the host species
Source: Chromosome Res. 2022 Jul 4;30(4):335–49. doi: 10.1007/s10577-022-09704-6 (PMC9771852; doi:10.1007/s10577-022-09704-6)

Supplemental Dataset 2


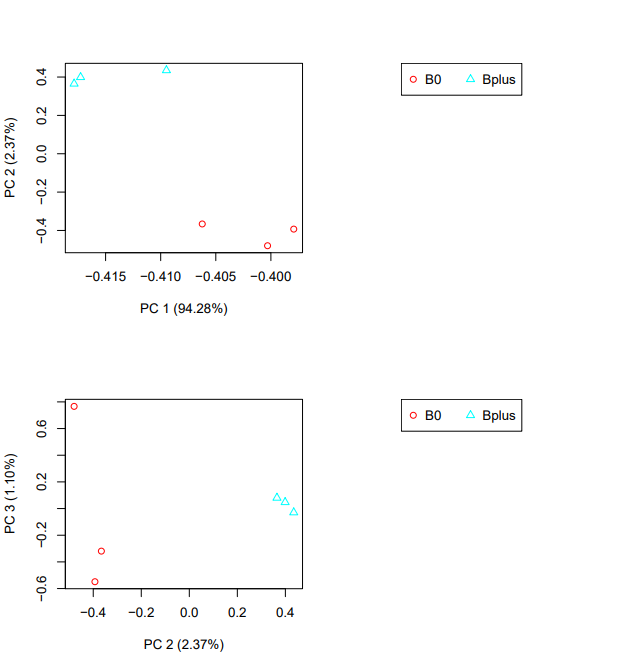
PCA plots based on the “PtR” of the Trinity pipeline. B0 (in red) represents three biological replicates of rye samples without Bs, Bplus (light blue) are biological replicates representing rye with +2Bs.

Pearson’s replicate correlation heatmap. A) Biological replicates of rye without Bs are called B0; B) Biological replicates of rye with +2Bs are called Bplus

A)


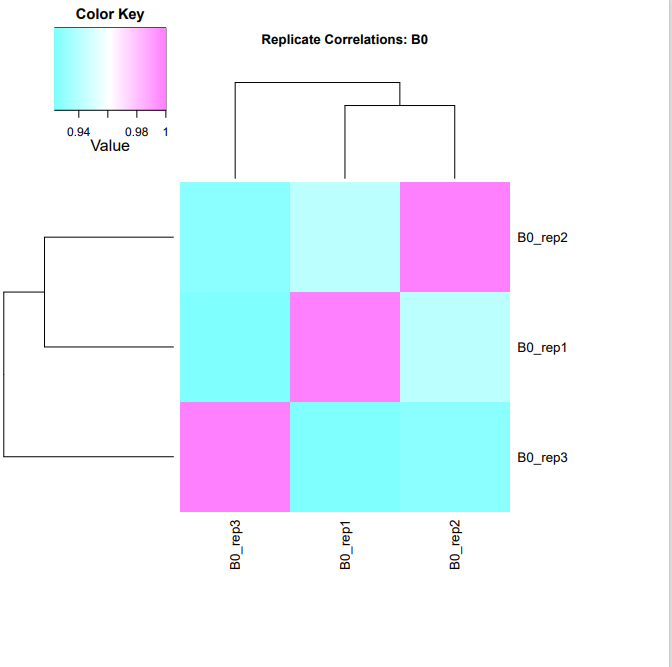


B)


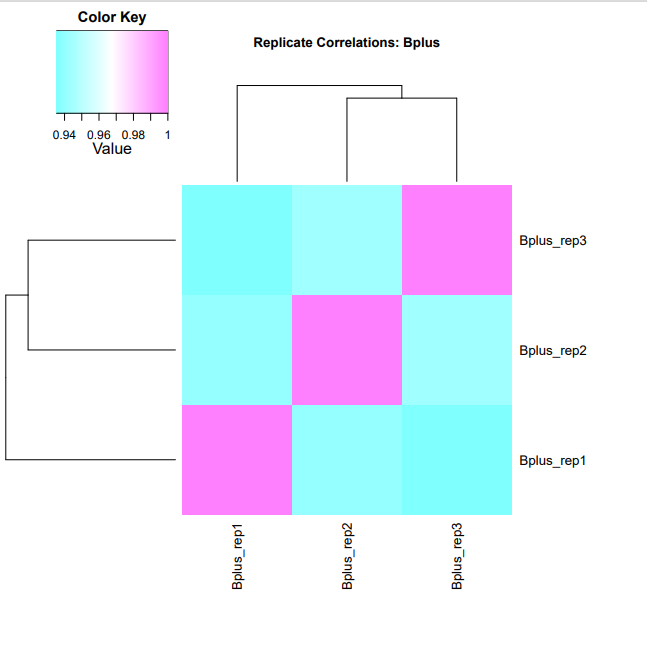

Supplement: Supplementary file 2 — Supplemental Dataset 2 PCA plots based on the “PtR” of the Trinity pipeline. Pearson’s replicate correlation heatmap. (DOCX 88 KB) [file 10577_2022_9704_MOESM2_ESM.docx]
