# Supplementary material for: Rye B chromosomes differently influence the expression of A chromosome–encoded genes depending on the host species
Source: Chromosome Res. 2022 Jul 4;30(4):335–49. doi: 10.1007/s10577-022-09704-6 (PMC9771852; doi:10.1007/s10577-022-09704-6)

Supplemental Dataset 3

Correlation of four biological replicates of rye B specific transcripts derived from wheat +2B plants.


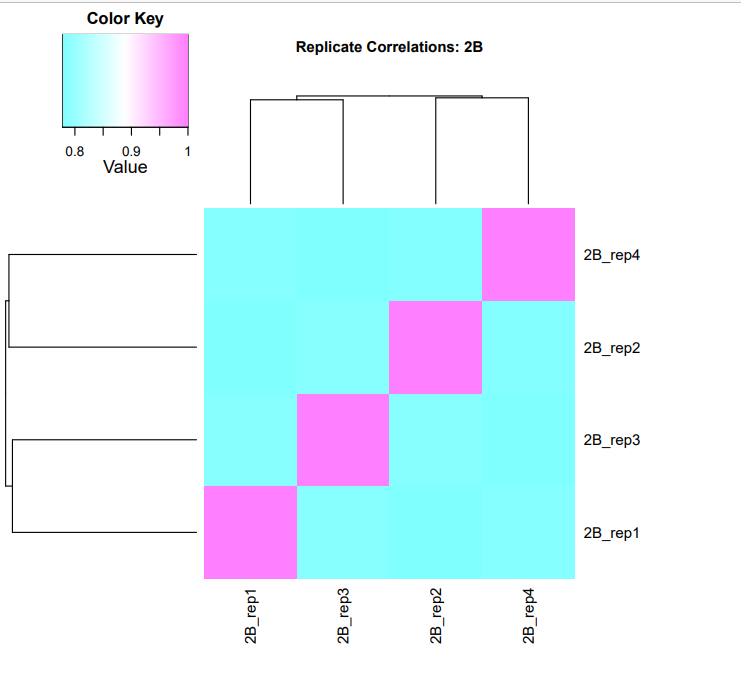


Correlation of four biological replicates of rye B specific transcripts derived from wheat +4B plants


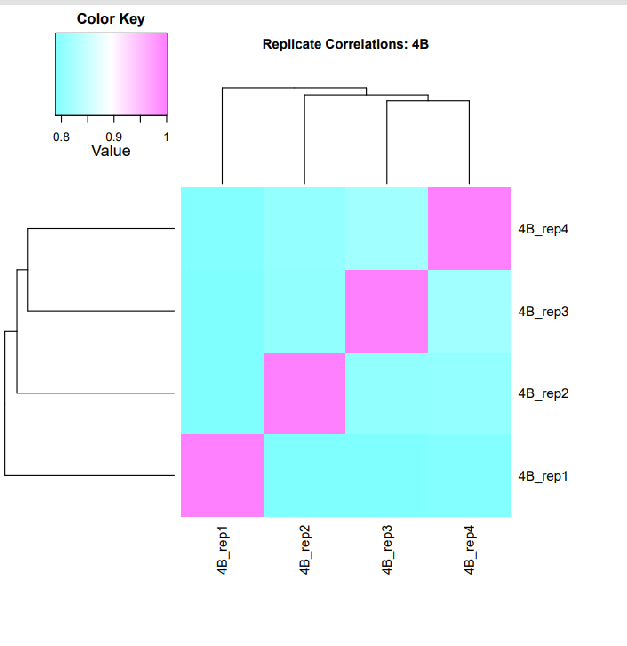


PCA based on four biological replicates of rye B specific transcripts derived from wheat +2B and wheat +4B plants


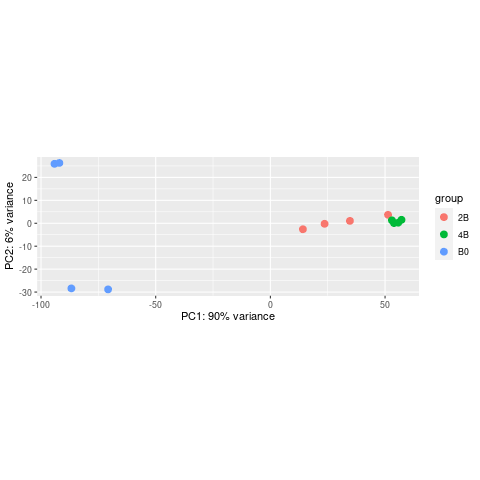

Supplement: Supplementary file 3 — Supplemental Dataset 3 Correlation of four biological replicates of rye B specific transcripts derived from wheat +2B plants. Correlation of four biological replicates of rye B specific transcripts derived from wheat +4B plants. PCA based on four biological replicates of rye B specific transcripts derived from wheat +2B and wheat +4B plants. (DOCX 73 KB) [file 10577_2022_9704_MOESM3_ESM.docx]
