# Supplementary material for: Rye B chromosomes differently influence the expression of A chromosome–encoded genes depending on the host species
Source: Chromosome Res. 2022 Jul 4;30(4):335–49. doi: 10.1007/s10577-022-09704-6 (PMC9771852; doi:10.1007/s10577-022-09704-6)

Supplemental Fig. 1

Correlation of three biological replicates of rye B specific transcripts derived from rye +2B plants.


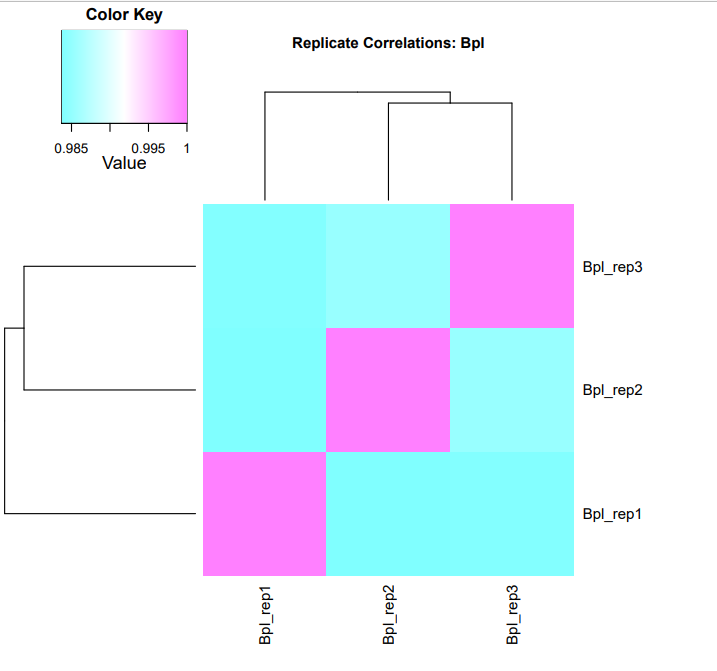

Supplement: Supplementary file 8 — Supplementary file8 (DOCX 36 KB) [file 10577_2022_9704_MOESM8_ESM.docx]
