# Supplementary figures and images for: Rye B chromosomes differently influence the expression of A chromosome–encoded genes depending on the host species
Source: Chromosome Res. 2022 Jul 4;30(4):335–49. doi: 10.1007/s10577-022-09704-6 (PMC9771852; doi:10.1007/s10577-022-09704-6)

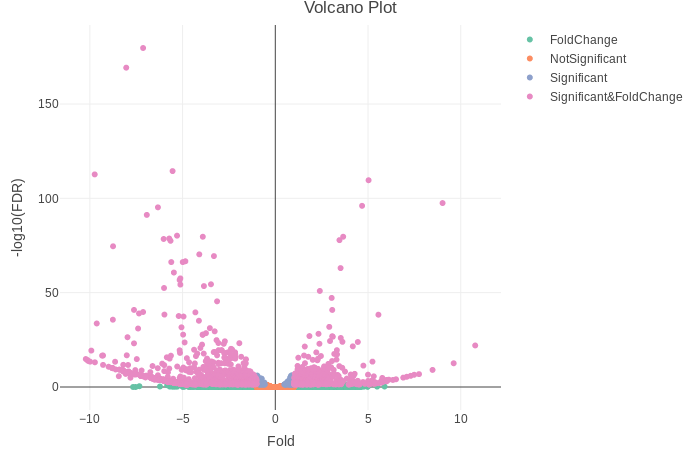

Supplement: Supplementary file 9 — Supplementary file9 (PNG 31 KB) [file 10577_2022_9704_MOESM9_ESM.png]

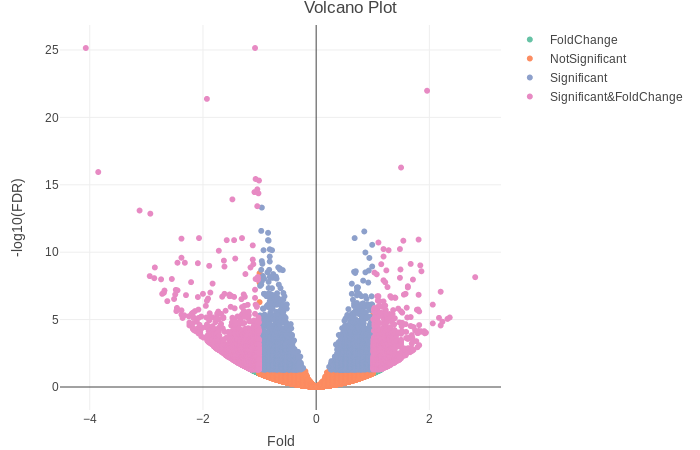

Supplement: Supplementary file 10 — Supplementary file10 (PNG 38 KB) [file 10577_2022_9704_MOESM10_ESM.png]
