# Supplementary material for: Rye B chromosomes differently influence the expression of A chromosome–encoded genes depending on the host species
Source: Chromosome Res. 2022 Jul 4;30(4):335–49. doi: 10.1007/s10577-022-09704-6 (PMC9771852; doi:10.1007/s10577-022-09704-6)
Supplement: Supplementary file 11 — Supplementary file11 (PPTX 620 KB) [file 10577_2022_9704_MOESM11_ESM.pptx]

## Slide 1
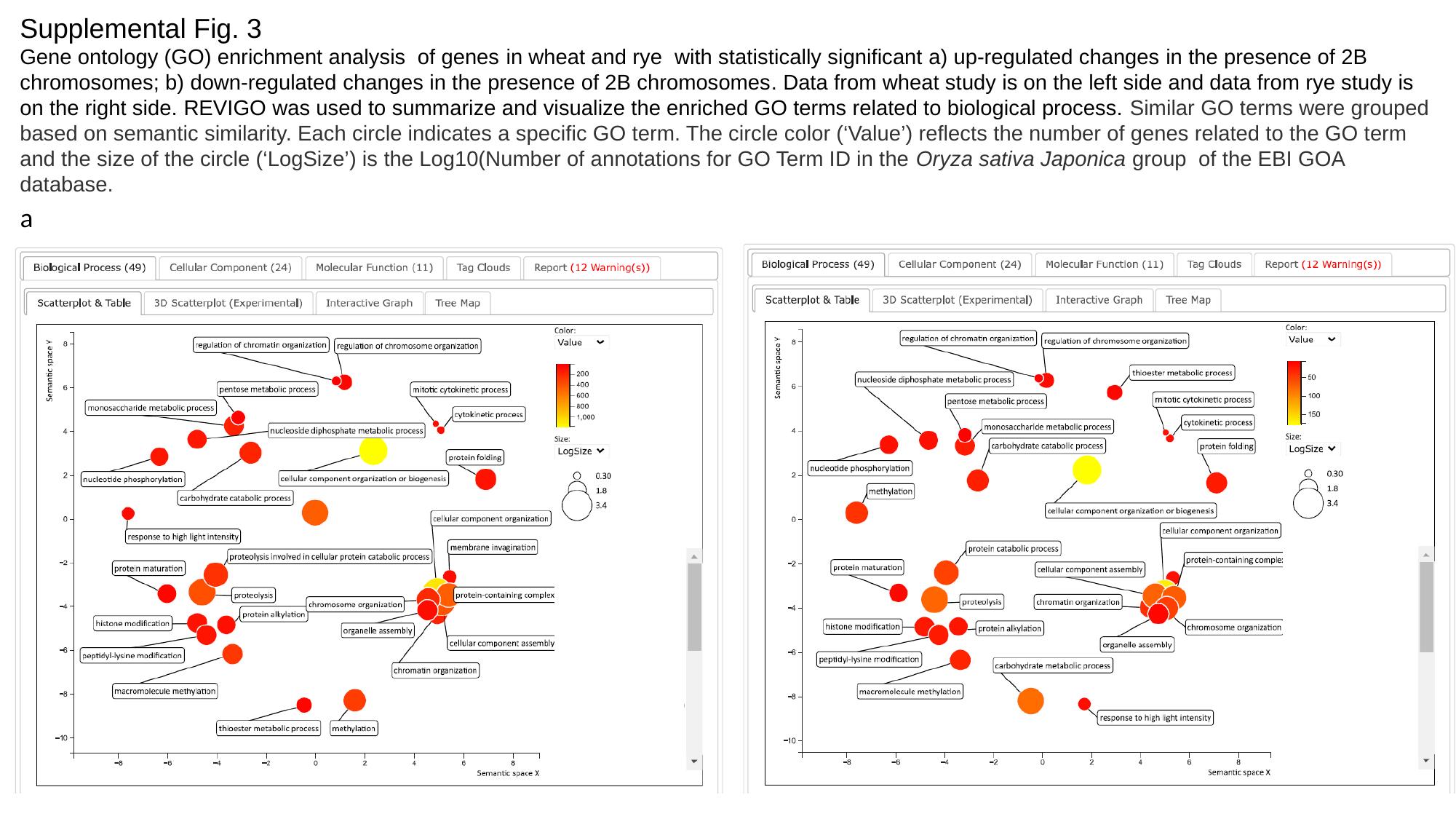

Supplemental Fig. 3
Gene ontology (GO) enrichment analysis of genes in wheat and rye with statistically significant a) up-regulated changes in the presence of 2B chromosomes; b) down-regulated changes in the presence of 2B chromosomes. Data from wheat study is on the left side and data from rye study is on the right side. REVIGO was used to summarize and visualize the enriched GO terms related to biological process. Similar GO terms were grouped based on semantic similarity. Each circle indicates a specific GO term. The circle color (‘Value’) reflects the number of genes related to the GO term and the size of the circle (‘LogSize’) is the Log10(Number of annotations for GO Term ID in the Oryza sativa Japonica group  of the EBI GOA database.
a

## Slide 2
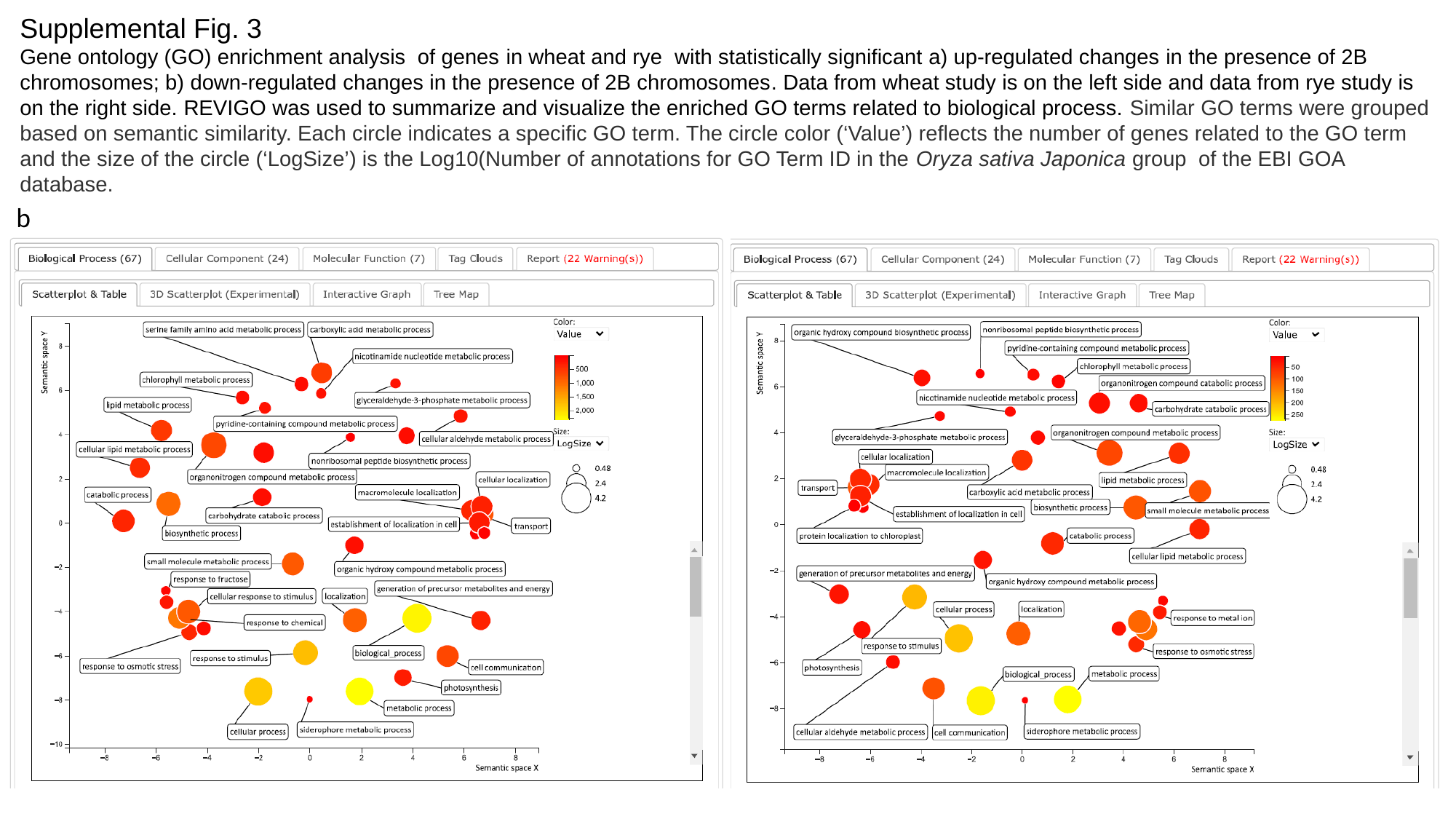

Supplemental Fig. 3
Gene ontology (GO) enrichment analysis of genes in wheat and rye with statistically significant a) up-regulated changes in the presence of 2B chromosomes; b) down-regulated changes in the presence of 2B chromosomes. Data from wheat study is on the left side and data from rye study is on the right side. REVIGO was used to summarize and visualize the enriched GO terms related to biological process. Similar GO terms were grouped based on semantic similarity. Each circle indicates a specific GO term. The circle color (‘Value’) reflects the number of genes related to the GO term and the size of the circle (‘LogSize’) is the Log10(Number of annotations for GO Term ID in the Oryza sativa Japonica group  of the EBI GOA database.
b
