# Supplementary material for: Rye B chromosomes differently influence the expression of A chromosome–encoded genes depending on the host species
Source: Chromosome Res. 2022 Jul 4;30(4):335–49. doi: 10.1007/s10577-022-09704-6 (PMC9771852; doi:10.1007/s10577-022-09704-6)
Supplement: Supplementary file 12 — Supplementary file12 (PPTX 71 KB) [file 10577_2022_9704_MOESM12_ESM.pptx]

## Slide 1
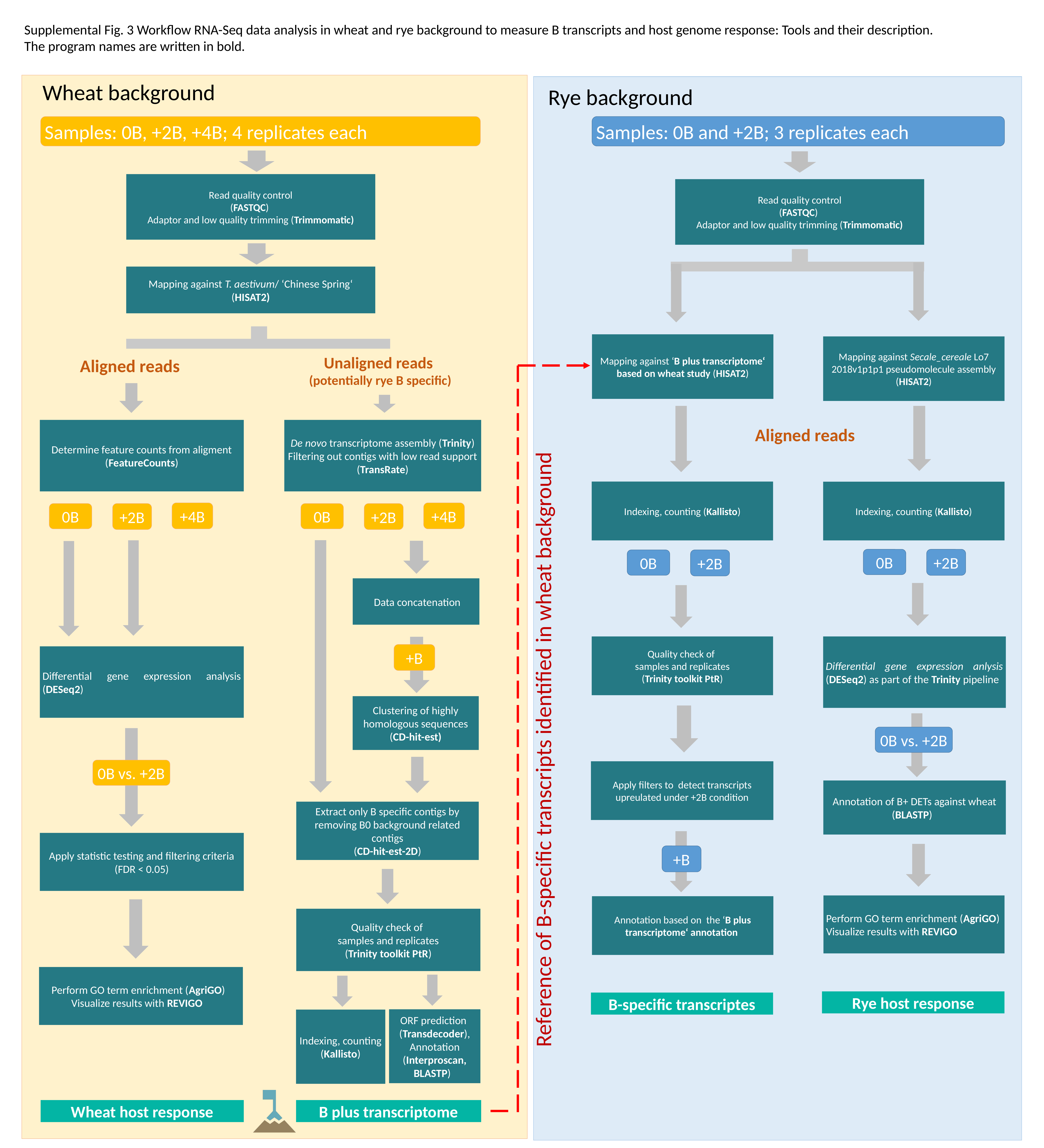

Supplemental Fig. 3 Workflow RNA-Seq data analysis in wheat and rye background to measure B transcripts and host genome response: Tools and their description.
The program names are written in bold.
Wheat background
Rye background
Samples: 0B and +2B; 3 replicates each
Samples: 0B, +2B, +4B; 4 replicates each
Read quality control
(FASTQC)
Adaptor and low quality trimming (Trimmomatic)
Read quality control
(FASTQC)
Adaptor and low quality trimming (Trimmomatic)
Mapping against T. aestivum/ ‘Chinese Spring‘ (HISAT2)
Mapping against ‘B plus transcriptome‘ based on wheat study (HISAT2)
Mapping against Secale_cereale Lo7 2018v1p1p1 pseudomolecule assembly (HISAT2)
Unaligned reads
(potentially rye B specific)
Aligned reads
Determine feature counts from aligment (FeatureCounts)
De novo transcriptome assembly (Trinity)
Filtering out contigs with low read support (TransRate)
Aligned reads
Indexing, counting (Kallisto)
Indexing, counting (Kallisto)
+4B
+4B
0B
+2B
0B
+2B
0B
+2B
0B
+2B
 Data concatenation
Quality check of
samples and replicates
(Trinity toolkit PtR)
Differential gene expression anlysis (DESeq2) as part of the Trinity pipeline
+B
Differential gene expression analysis (DESeq2)
Clustering of highly homologous sequences
(CD-hit-est)
0B vs. +2B
Reference of B-specific transcripts identified in wheat background
0B vs. +2B
Apply filters to detect transcripts upreulated under +2B condition
Annotation of B+ DETs against wheat (BLASTP)
Extract only B specific contigs by removing B0 background related contigs
(CD-hit-est-2D)
Apply statistic testing and filtering criteria (FDR < 0.05)
+B
Perform GO term enrichment (AgriGO)
Visualize results with REVIGO
Annotation based on the ‘B plus transcriptome‘ annotation
Quality check of
samples and replicates
(Trinity toolkit PtR)
 Perform GO term enrichment (AgriGO)
 Visualize results with REVIGO
Rye host response
B-specific transcriptes
ORF prediction (Transdecoder),
Annotation (Interproscan, BLASTP)
Indexing, counting
(Kallisto)
Wheat host response
B plus transcriptome
